# Reduced dorsal fronto-striatal connectivity at rest in anorexia nervosa

**DOI:** 10.1017/S003329172400031X

**Published:** 2024-07

**Authors:** Alexandra F. Muratore, Karin Foerde, E. Caitlin Lloyd, Caroline Touzeau, Blair Uniacke, Natalie Aw, David Semanek, Yun Wang, B. Timothy Walsh, Evelyn Attia, Jonathan Posner, Joanna E. Steinglass

**Affiliations:** 1 Department of Psychiatry, Columbia University Irving Medical Center, New York, NY, USA; 2 New York State Psychiatric Institute, New York, NY, USA; 3 Department of Psychology, University of Amsterdam, Amsterdam, The Netherlands; 4 Department of Psychiatry, Duke University, Durham, NC, USA

**Keywords:** anorexia nervosa, anterior caudate, dorsal fronto-striatal circuitry, fMRI, repetitive transcranial magnetic stimulation, resting-state functional connectivity

## Abstract

**Background:**

Anorexia nervosa (AN) is a serious psychiatric illness that remains difficult to treat. Elucidating the neural mechanisms of AN is necessary to identify novel treatment targets and improve outcomes. A growing body of literature points to a role for dorsal fronto-striatal circuitry in the pathophysiology of AN, with increasing evidence of abnormal task-based fMRI activation within this network among patients with AN. Whether these abnormalities are present at rest and reflect fundamental differences in brain organization is unclear.

**Methods:**

The current study combined resting-state fMRI data from patients with AN (*n* = 89) and healthy controls (HC; *n* = 92) across four studies, removing site effects using ComBat harmonization. First, the *a priori* hypothesis that dorsal fronto-striatal connectivity strength – specifically between the anterior caudate and dlPFC – differed between patients and HC was tested using seed-based functional connectivity analysis with small-volume correction. To assess specificity of effects, exploratory analyses examined anterior caudate whole-brain connectivity, amplitude of low-frequency fluctuations (ALFF), and node centrality.

**Results:**

Compared to HC, patients showed significantly reduced right, but not left, anterior caudate-dlPFC connectivity (*p* = 0.002) in small-volume corrected analyses. Whole-brain analyses also identified reduced connectivity between the right anterior caudate and left superior frontal and middle frontal gyri (*p* = 0.028) and increased connectivity between the right anterior caudate and right occipital cortex (*p* = 0.038). No group differences were found in analyses of anterior caudate ALFF and node centrality.

**Conclusions:**

Decreased coupling of dorsal fronto-striatal regions indicates that circuit-based abnormalities persist at rest and suggests this network may be a potential treatment target.

## Introduction

Anorexia nervosa (AN) is a serious psychiatric disorder characterized by extreme restriction of food intake, fear or avoidance of weight gain, and persistent body image disturbance (American Psychiatric Association, 2013). It is associated with severe medical and psychiatric symptoms, substantial social and economic burdens, and high mortality rates (Arcelus, Mitchell, Wales, & Nielsen, 2011; Gibson, Workman, & Mehler, 2019; Mehler & Brown, 2015). Current behavioral and pharmacological treatments are disappointing, with low rates of recovery and high rates of relapse (Berends et al., 2016; Khalsa, Portnoff, McCurdy-McKinnon, & Feusner, 2017). The significant burden of AN and the limitations of available treatments highlight the need for a better understanding of underlying neural mechanisms of illness. Examination of the brain at rest, absent task demands, and performance differences between groups, may be particularly valuable for identifying neural circuit abnormalities and clarifying which brain regions and networks may prove useful as novel treatment targets.

Neural systems linking the frontal cortex and the striatum are engaged during executive function processes that guide human behavior in a healthy brain (Alexander, DeLong, & Strick, 1986; Haber & Knutson, 2010). Altered connectivity within these fronto-striatal circuits has been linked to a range of psychiatric disorders (Burguière, Monteiro, Mallet, Feng, & Graybiel, 2015; Furman, Hamilton, & Gotlib, 2011; Kim et al., 2019; Morein-Zamir & Robbins, 2015). The dorsal fronto-striatal system includes connections between the dorsolateral prefrontal cortex (dlPFC) and the dorsal striatum, consisting of the caudate and putamen (Alexander et al., 1986; Morris et al., 2016), and is thought to mediate cognitive processes such as inhibitory control, goal-directed behavior, reward responsivity, and cognitive flexibility, that are potentially clinically relevant in AN (Grahn, Parkinson, & Owen, 2008; Ojha, Parr, Foran, Calabro, & Luna, 2022; Staudinger, Erk, & Walter, 2011; Vaghi et al., 2017). Indeed, findings from MRI studies provide converging evidence of abnormalities within dorsal fronto-striatal regions among patients with AN, both structurally and across a variety of tasks (Foerde et al., 2020; Foerde, Steinglass, Shohamy, & Walsh, 2015; Foerde et al., 2021; Frank, Shott, Hagman, & Mittal, 2013; Friederich et al., 2012; Martin Monzon et al., 2017; Sanders et al., 2015; Titova, Hjorth, Schiöth, & Brooks, 2013; Zhu et al., 2012). Further, initial studies of the neural mechanisms of restrictive eating have found associations between the anterior region of the caudate – and, in one study, its connections to the dlPFC – with maladaptive restrictive food choices in AN (Foerde et al., 2015; Foerde et al., 2020; Foerde et al., 2021). Whether these abnormalities are reflective of a persistent disturbance (i.e. present at rest) in dorsal fronto-striatal connectivity among patients with AN remains unclear.

Resting-state fMRI (rsfMRI) measures low-frequency fluctuations in blood oxygenation level dependent (BOLD) signal across the brain in the absence of task or stimuli (Lee, Smyser, & Shimony, 2013; Smitha et al., 2017). By identifying temporal correlations between regions, rsfMRI characterizes the functional architecture of the brain at rest. RsfMRI is increasingly used to identify individualized targets for brain stimulation, such as repetitive transcranial magnetic stimulation (rTMS; Fox, Halko, Eldaief, and Pascual-Leone, 2012b; Fox, Liu, and Pascual-Leone, 2013). These targets are often within the dlPFC, due to the ease of access with non-invasive techniques, and connections between dlPFC and subcortical regions such as the dorsal striatum (Hanlon et al., 2013; Hanlon et al., 2015; Hanlon, Dowdle, & Henderson, 2018). Investigating resting-state functional connectivity between the dlPFC and the anterior caudate may be particularly useful for treatment development in AN due to these connections and the potential centrality of the anterior caudate in maladaptive eating behavior.

Existing rsfMRI studies of patients with AN are limited by small sample sizes, with an average sample size of approximately 27 per group. The largest rsfMRI study of AN, barring a meta-analysis (Su et al., 2021) and systematic review (Gaudio, Wiemerslage, Brooks, & Schiöth, 2016), examined data from 74 patients and 74 HC, though this study investigated amplitude of low frequency fluctuations (ALFF), a metric of BOLD signal intensity, and regional homogeneity, rather than functional connectivity of brain regions (Seidel et al., 2019). To maximize sample size and increase the reliability of findings, the current study combined data across four samples to obtain the largest dataset of rsfMRI among individuals with AN to date.

The primary aim of this study was to examine connection strength in dorsal fronto-striatal circuitry among patients with AN at rest. Seed-based functional connectivity analyses with small-volume correction were conducted to test the *a priori* hypothesis that connection strength between the anterior caudate and dlPFC would differ among patients with AN compared to healthy controls (HC). Hypotheses were non-directional as research on task-based functional connectivity between these regions has found both hyper and hypo-connectivity among patients with AN depending on task demands (Foerde et al., 2015).

To further examine the specificity of dorsal fronto-striatal connectivity differences, we explored whole-brain connectivity and regional BOLD signal of the anterior caudate at rest by comparing patients and controls on measures of seed-based functional connectivity, regional intensity (amplitude of low-frequency fluctuations [ALFF]), node centrality (intrinsic connectivity contrast [IC], and global correlation [GCOR]). Finally, among patients with AN, we explored relationships between rsfMRI measures and clinical symptoms.

## Materials and methods

### Procedures

The current study includes data from 181 participants (89 patients with AN, 92 HC), combined from four studies (Cha et al., 2016; Foerde et al., 2015; Foerde et al., 2020; Uniacke et al., 2019). RsfMRI data from two studies were reported previously (Cha et al., 2016; Uniacke et al., 2019), but did not include analyses of dorsal fronto-striatal circuits. RsfMRI data from the remaining two studies (Foerde et al., 2015; Foerde et al., 2020) have not been previously analyzed. All studies were approved by the NYSPI Institutional Review Board. Prior to participation, adults provided written informed consent and adolescents provided assent with parental consent.

#### Participants

All participants were female, between the ages of 14 and 40 years, not pregnant, with estimated IQ > 80 and without MRI contraindications. For individuals who participated in multiple studies, data from only one study was used. Across all studies, patients met DSM-5 (American Psychiatric Association, 2013) criteria for current AN and were excluded if they met criteria for current substance use disorder, lifetime psychotic disorder, or other major neurological disorders. Patients were free from psychotropic medications, except from one patient taking an antidepressant and were medically stable. HC had a BMI within the normal range (18.5–25 kg/m^2^) and were excluded if they met criteria for any current or past psychiatric diagnoses. Diagnosis of AN and presence of co-occurring psychiatric disorders were confirmed using Eating Disorders Assessment for DSM-5 (EDA-5; Sysko et al., 2015) and Structured Clinical Interview for DSM-5 (SCID-5; First, Williams, Karg, and Spitzer, 2015). Height and weight were measured by stadiometer and Detecto scale, respectively, and used to calculate BMI (kg/m^2^). Two studies obtained estimated IQ from the Wechsler Abbreviated Scale for Intelligence, 2nd edition (WASI-II; Wechsler, 2011); and two obtained estimated IQ from the Wechsler Test of Adult Reading (WTAR; Venegas and Clark, 2011) for individuals 16 and older. Standardized norms for the WTAR are not available for the six individuals under age 16, and IQ data were missing from an additional six participants.

#### Clinical assessments

Across all studies, psychological features of AN were assessed via the Eating Disorder Examination-Questionnaire (EDE-Q; Fairburn and Beglin, 2008) Global Score, and self-reported duration of illness. Two of the four studies also assessed actual eating behavior via calorie and percent fat intake during a buffet-style meal, using previously validated procedures (Sysko, Steinglass, Schebendach, Mayer, & Walsh, 2018).

#### MRI acquisition

Anatomical and resting-state fMRI scans were collected from all participants. For all studies, participants were instructed to ‘let their mind wander freely’ during the resting-state fMRI scans. Three studies instructed participants to keep their eyes open during the scan; one study instructed participants to keep their eyes closed. See online Supplementary Table S1 for the resting-state scanning sequence parameters in each of the four studies.

### Statistical analyses

#### Participant demographics

Demographic and clinical variables were compared between diagnostic groups with independent-samples *t* tests using SPSS version 28.

#### MRI preprocessing

MRI data were preprocessed and analyzed using the CONN toolbox version 21a (Whitfield-Gabrieli & Nieto-Castanon, 2012). Preprocessing steps included realignment and unwarping, slice-timing correction, outlier identification, normalization, and smoothing with an 8 mm smoothing kernel full width at half maximum. Time series data were denoised using linear regression of potential confounds, including fMRI signal from white matter and cerebrospinal fluid, subject-motion parameters (three translation, three rotation, and associated first-order derivatives), outliers, and temporal band-pass filtering (0.01 to Inf). Outliers were volumes with excessive movement, defined as framewise displacement (FD) >0.5 mm or global signal intensity changes (GSC) >3 s.d. using the artifact detection tool (ART^1^). Outliers were indexed with nuisance regressors (motion corrupted volume and volume preceding it) and runs with >25% outlier volumes were excluded from analyses. Six runs (3 AN, 3 HC) with >25% outlier volumes were excluded from analyses. No group differences in head motion were detected (see online Supplementary Table S2 for mean FD/GSC). For participants with rsfMRI data from multiple runs, data from each preprocessed, denoised run were concatenated for first and second-level analyses.

#### Regions of interest (ROIs)

**Figure 1.**
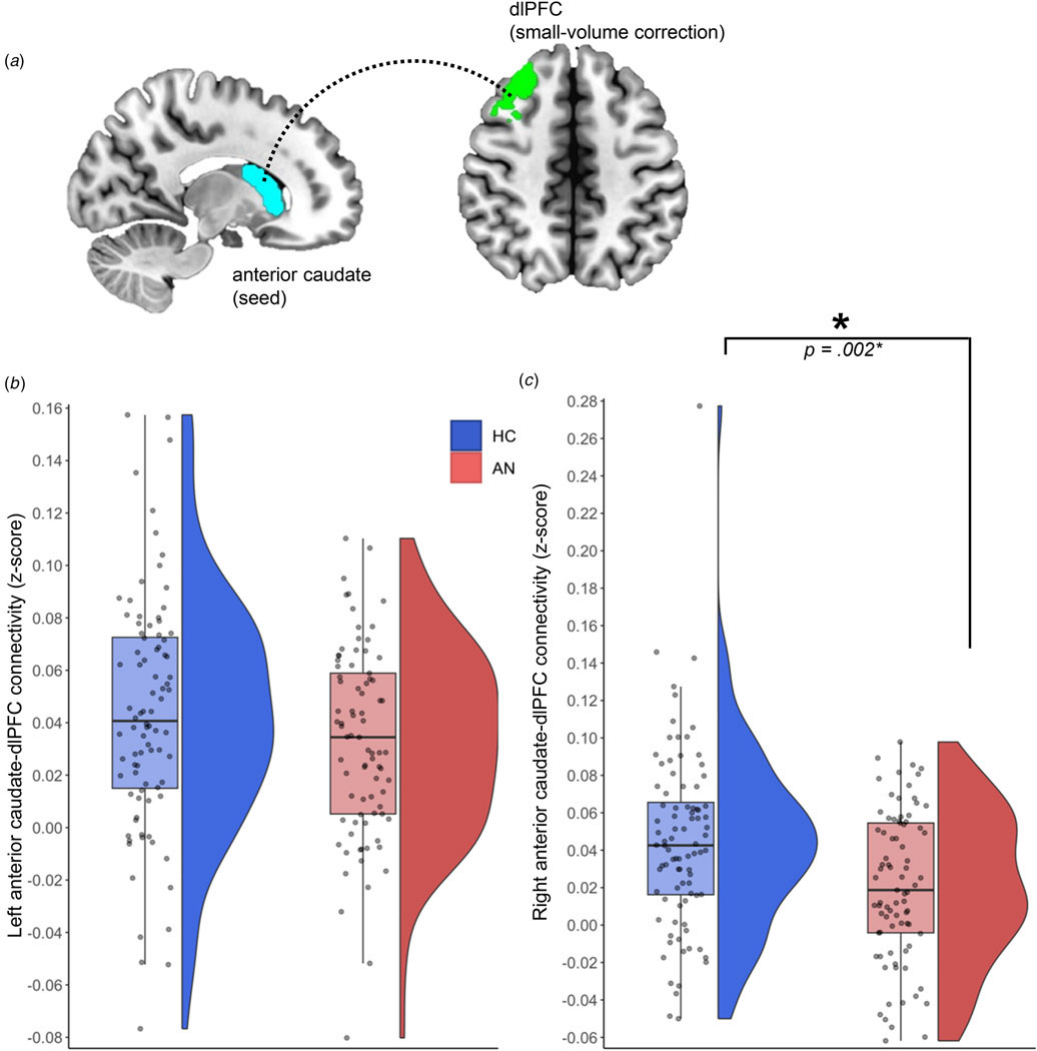
Results of seed-based analysis comparing left and right anterior caudate-dlPFC connectivity between HC and individuals with AN. (a) Anterior caudate seed (light blue) used in functional connectivity analyses with a small volume correction within the ipsilateral dlPFC target region (green). (b) No significant group differences in left anterior caudate-dlPFC connectivity. (c) Group difference in right anterior caudate-dlPFC connectivity; compared to HC, individuals with AN exhibit significantly reduced connectivity between the right anterior caudate and voxels within the right dlPFC target region. *Group difference remains significant when removing one outlier from HC group, *p* = 0.002.

Four ROIs were selected for use in analyses: left and right anterior caudate, and left and right dlPFC (Fig. 1a). As in prior publications (Foerde et al., 2015; Foerde et al., 2020; Foerde et al., 2021), the left and right anterior caudate ROIs were defined using masks for the left and right caudate from the Harvard-Oxford Atlas maximum-likelihood subcortical atlas included in FSL (Jenkinson, Beckmann, Behrens, Woolrich, & Smith, 2012). These seeds were thresholded at 25% probability and further parcellated to isolate their anterior portion by only including the portion of the mask anterior to y = 0. The dlPFC ROIs were defined using Brodmann's areas 9/46d and 9/46d based on the Sallet Dorsal Frontal Connectivity-Based Parcellation Atlas (Sallet et al., 2013).

#### Data harmonization

Because data in the current study were collected across several MRI scanners, inter-site variability may arise from differences in scanner acquisitions. Therefore, fMRI data were harmonized using ComBat, a validated methodology designed to reduce variability related to differences in MRI acquisition, while preserving biological variability (Fortin et al., 2018; Fortin et al., 2017; Yamashita et al., 2019; Yu et al., 2018). Parameter estimates from each analysis were extracted and harmonized using the CombatHarmonization package in R Studio (R Studio Team, 2020). To control for site effects, the study site for each participant was entered into the model. Participant age, BMI, IQ, and group (AN *v.* HC) were included to preserve these factors as sources of biological variability.

### Hypothesis testing

#### Seed-based functional connectivity

Seed-based functional connectivity analyses were conducted using the left and right anterior caudate as seeds. Mean time series from each seed was correlated with all voxels in the brain, thereby producing two whole-brain functional connectivity maps per participant. Whole-brain connectivity maps were Fisher-transformed and extracted for harmonization, following procedures described above. Harmonized anterior caudate connection strength was then compared between diagnostic groups. Statistical significance was determined with voxel-wise height threshold of *p*-uncorrected <0.001 and cluster-size *p*-FDR-corrected threshold *p* < 0.05. To test the primary hypothesis that patients with AN would exhibit differences in dorsal fronto-striatal connectivity, small-volume correction was employed to restrict the analysis to the DLPFC ROIs. Group differences were tested using general linear models (GLMs), with ipsilateral connection strength as the dependent variable and group as the independent variable, while controlling for age and IQ. Small-volume correction analyses were repeated with unharmonized data, with the addition of site as a control variable, as well as a group-by-site interaction term. To explore additional group differences in anterior caudate connectivity, whole-brain analyses without small-volume correction were performed on harmonized and unharmonized functional connectivity maps using the same GLMs as described above.

### Exploratory MRI analyses

To further explore whether potential group differences in anterior caudate-DLPFC connectivity may be indicative of group differences in localized activity or globally reduced connection to other brain regions, we conducted two additional exploratory fMRI analyses using the CONN toolbox (Nieto-Castanon, 2020; Whitfield-Gabrieli et al., 2016): *Anterior Caudate Regional Intensity, and Anterior Caudate Node Centrality.* Statistical significance was determined with a height threshold of *p*-uncorrected <0.001 and cluster-size *p*-FDR-corrected threshold *p* < 0.05.

#### Anterior caudate regional intensity

Previously identified group differences in anterior caudate activity (Foerde et al., 2015; Foerde et al., 2020; Foerde et al., 2021) and connectivity (Foerde et al., 2015) during a food choice task may also be reflective of a persistent, baseline disturbance in anterior caudate activity, such that patients with AN may have hypo- or hyper-activation of this brain region, regardless of task. ALFF is defined as the root mean square of BOLD signal within each voxel following band-pass filtering, and is considered a measure of baseline regional intensity of BOLD signal in a given region (Zang et al., 2007). To further characterize the activity of the anterior caudate at rest, whole-brain ALFF was calculated and harmonized. Harmonized ALFF values within the anterior caudate ROIs were then compared between groups, with ALFF within each region as the dependent variable and group as the independent variable, while controlling for age and IQ. Analyses were repeated with unharmonized data, with the addition of site as a control variable, as well as a group-by-site interaction term.

#### Anterior caudate node centrality

We explored node-level centrality of the anterior caudate at rest using intrinsic connectivity contrast (IC) and global correlation (GCOR). IC and GCOR provide measures of node centrality, i.e. the mean connection strength of a given voxel with all other voxels across the brain. IC is defined as the root mean square of correlation coefficients between the ROI and all other voxels, whereas GCOR is calculated using the mean correlation strength between the ROI and all other voxels (Martuzzi et al., 2011; Nieto-Castanon, 2020). Whole-brain IC and GCOR were calculated and harmonized. Harmonized IC and GCOR values within the anterior caudate ROIs were then extracted and compared between groups, with each measure of node centrality as the dependent variable and group as the independent variable, while controlling for age and IQ. Analyses were repeated with unharmonized data, with the addition of site as a control variable, as well as a group-by-site interaction term.

#### Clinical correlates

Partial correlations controlling for age and IQ were conducted to explore associations between regions with group differences in connection strength and clinical variables within the AN group, including psychological features of AN (measured via Global EDE-Q scores), duration of illness and, among a subsample of participants (*n* = 45), restrictive eating behavior (measured via caloric and percent fat intake during a buffet-style meal). Multiple comparisons were controlled for using Bonferroni corrections.

## Results

### Participant demographics

A total of 181 resting-state scans from 89 AN (54% binge-eating/purging subtype) and 92 HC were collected. Demographic and clinical characteristics of participants are presented in Table 1, for each study separately and for the full combined sample. As anticipated, BMI was significantly lower and psychological symptoms of AN, as measured by the EDE-Q Global score, were significantly higher among patients with AN than HC. Scatterplots representing the distribution and range of clinical characteristics of the patient sample (BMI, EDE-Q Global scores, Duration of Illness) are included in online Supplementary Figure S1. Because IQ was included in the harmonization model as a source of biological variability, the results of harmonized analyses among 169 participants with complete data are presented below. Analyses of unharmonized data with (*n* = 169) and without (*n* = 181) IQ as a covariate yielded a similar pattern of results and are presented in the Supplement.

**Table 1.** Participant Characteristics, for combined samples and each study separately

	Combined			Study							
				1		2		3		4	
Group	HC	AN		HC	AN	HC	AN	HC	AN	HC	AN
*n*	92	89		26	24	13	16	36	31	17	18
Age	22.42 (4.83)	23.30 (6.25)		19.19 (2.91)	19.54 (3.51)	22.08 (2.47)	26.88 (6.93)	25.75 (5.32)	26.58 (6.23)	20.59 (2.76)	19.50 (2.75)
IQ	112.58 (11.22)	107.70 (9.28)	**	108.63 (9.00)	103.56 (10.93)	113.54 (8.69)	108.00 (8.53)	116.56 (12.53)	111.07 (8.96)	108.53 (10.34)	105.51 (8.72)
EDE-Q	0.23 (0.35)	4.23 (1.37)	**	0.15 (0.20)	4.16 (1.26)	0.06 (0.08)	4.38 (1.15)	0.30 (0.47)	4.27 (1.49)	0.07 (0.14)	3.93 (1.58)
BMI	21.22 (1.75)	16.38 (1.85)	**	21.04 (1.69)	16.29 (1.95)	21.51 (2.33)	15.73 (2.17)	21.03 (1.59)	16.28 (1.77)	21.69 (1.71)	17.25 (1.24)

### Hypothesis testing

#### Seed-based functional connectivity

Results showed no significant effect of group for left anterior caudate-left dlPFC connectivity, *F*[1167] = 2.51, *p* = 0.115, *η*p^2^ = 0.02. A significant effect of group was observed for right anterior caudate-right dlPFC connectivity (*F*[1169] = 9.45, *p* = 0.002, *η*p^2^ = 0.05), such that individuals with AN had reduced mean connection strength (*M* = 0.02, *SE* = 0.004) as compared with HC (*M* = 0.05, *SE* = 0.005; Figure 1; Table S3). The same pattern of results was obtained with unharmonized data, with a significant effect of group for right, but not left, anterior caudate-dlPFC connectivity; group-by-site interactions were non-significant (see online Supplementary Figure S4, S6 and Tables S5, S9). Unthresholded, whole-brain maps of these seed-based analyses using harmonized and unharmonized data can be found at https://neurovault.org/collections/FWXXMIWD/.

Results of follow-up analyses with harmonized data indicated no significant difference between subtypes of AN (online Supplementary Figure S2). To determine whether psychiatric comorbidities among patients with AN affected results, we conducted a sensitivity analysis comparing HC and AN patients with no psychiatric comorbidities, and observed the same pattern of results (online Supplementary Figure S3).

### Exploratory MRI analyses

#### Whole-Brain functional connectivity

**Figure 2.**
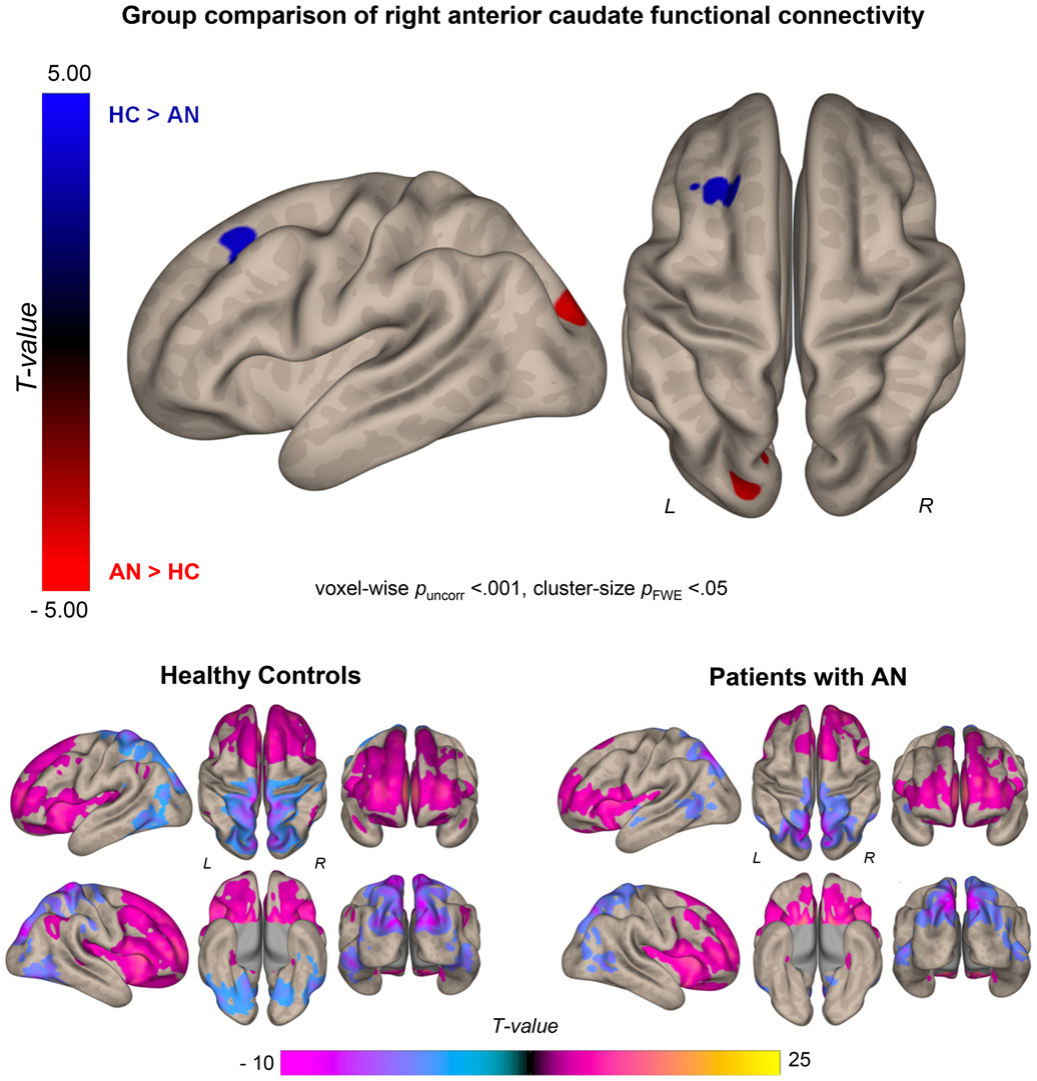
Top row: Comparison of whole-brain connectivity with the right anterior caudate between HC and individuals with AN (AN > HC = red; HC > AN = blue). Compared to HC, individuals with AN exhibit hypoconnectivity between the right anterior caudate and bilateral superior frontal gyrus/left middle frontal gyrus and hyperconnectivity between the right anterior caudate and bilateral occipital cortex. Bottom row: right anterior caudate functional connectivity maps among HC (left) and patients with AN (right).

Results of exploratory whole-brain analyses identified a significant effect of group for right anterior caudate connectivity with the superior frontal gyrus (SFG) and occipital cortex (Fig. 2, online Supplementary Table S4): compared to HC, individuals with AN exhibited decreased connectivity between the right anterior caudate and the left SFG, and increased connectivity between the right anterior caudate and left occipital cortex. There was no significant effect of group for left anterior caudate connectivity. Seed-based functional connectivity analyses with unharmonized data also identified reduced right anterior caudate-left SFG connectivity, with additional findings of group differences in left and right anterior caudate connectivity with the insula, hippocampus, and supramarginal gyrus. Group-by-site interactions were not significant (see online Supplementary Figures S5, S7 and Tables S6, S10).

#### Anterior caudate regional intensity

There were no significant effects of group on ALFF for the left or right anterior caudate (Table 2). The same results were obtained with unharmonized data analyses, with no significant effects of group or group-by-site interactions. Results of statistical comparisons of ALFF between groups using harmonized and unharmonized data are included in online Supplementary Tables S7, S11.

**Table 2.** Anterior caudate regional intensity and node centrality and regional intensity analyses. General linear models examined effects of group (AN v. HC) controlling for age and IQ

			Left anterior caudate			Right anterior caudate		
Measure		Predictor	*F*	*p*	*η*^2^*p*	*F*	*p*	*η*^2^*p*
Regional intensity	Amplitude of low-frequency fluctuations (ALFF)	Group	0.54	0.464	<0.01	0.99	0.322	0.01
		Age	2.09	0.150	0.01	0.91	0.342	0.01
		IQ	0.001	0.979	0.00	0.41	0.521	<0.01
Node centrality	Intrinsic connectivity (IC)	Group	0.46	0.498	<0.01	0.46	0.500	<0.01
		Age	2.34	0.128	0.01	1.29	0.259	0.01
		IQ	1.13	0.289	0.01	0.59	0.444	<0.01
	Global correlation (GCOR)	Group	0.95	0.332	0.01	2.35	0.128	0.01
		Age	5.67	0.018	0.03	2.98	0.086	0.02
		IQ	0.46	0.498	<0.01	0.54	0.463	<0.01

#### Anterior caudate node centrality

There were no significant effects of group on IC or GCOR for left or right anterior caudate (Table 2). The same results were obtained with unharmonized data, with no significant effect of group and no significant group-by-site interaction. Results of statistical comparisons of IC and GCOR between groups using unharmonized data are included is online Supplementary Table S8, S12.

### Clinical correlates

Among patients with AN, no partial correlations survived correction for multiple comparisons. Summary of correlational findings are included in online Supplementary Table S13.

## Discussion

The present study combined data across four studies of individuals with AN and healthy peers to investigate differences in dorsal fronto-striatal circuits in the largest investigation of resting-state functional connectivity in AN to date, to our knowledge. Consistent with our *a priori* hypothesis, decreased connection strength between the right anterior caudate and the right dlPFC was found among patients with AN relative to HC. Results of exploratory whole-brain analyses suggested reduced connectivity between the right anterior caudate and left SFG/MFG, providing additional evidence of reduced dorsal fronto-striatal circuit connectivity among patients with AN.

Data on the dorsal fronto-striatal circuit among patients with AN are limited, though emerging research points to disturbances in connectivity within this circuit (Foerde et al., 2015; Haynos et al., 2019). Insights from task-based fMRI research indicate that connectivity within this circuit mediates several important functions in healthy individuals relevant to AN, including goal-directed behavior and decision-making (Heekeren, Marrett, & Ungerleider, 2008; Hutcherson & Tusche, 2022). Among patients with AN, certain research suggests that differential connectivity strength within this circuit may underlie restrictive decisions about food: during a food choice task, patients showed hyperconnectivity between the anterior caudate and dlPFC when making decisions about low-fat foods, but reduced connectivity between these regions when making decisions about high-fat foods, as compared with HC (Foerde et al., 2015). The current findings of reduced rsfMRI connection strength are consistent with that of a previous rsfMRI study, which found reduced connection strength between a bilateral dorsal caudate seed and frontal regions, including the SFG and orbitofrontal cortex (OFC), among 19 patients with AN-R (Haynos et al., 2019), and indicate that these network-level abnormalities persist even at rest. The association between functional connectivity at rest and connectivity when completing a task or confronted with disorder-relevant cues has not been fully explored in AN, and merits further investigation.

Identification of abnormalities within dorsal fronto-striatal connections among individuals with AN suggests this circuit may be a potential target for treatment. The dlPFC, in particular, is the brain target for rTMS in the treatment of other psychiatric disorders, such as major depressive disorder (MDD; O'Reardon et al., 2007). Initial studies of rTMS to the same dlPFC target used for MDD among patients with AN found modest improvement in symptoms (Dalton et al., 2018; 2020a; 2020b). A more recent study by our group found that compared to sham, rTMS to a region of the dlPFC previously implicated in food choice among inpatients with AN was associated with a significant reduction in fat avoidance, as measured by a food choice task (Muratore et al., 2021). The present findings of differences in right anterior caudate-dlPFC strength provide evidence to further support the use of the dlPFC as a potential brain stimulation target through which to modulate dorsal fronto-striatal circuitry among patients with AN. Given the substantial variability in intrinsic connectivity, rsfMRI data also enables selection of individualized cortical targets within the dlPFC region based on functional connectivity to subcortical regions of interest, which preliminary research suggests may improve rTMS treatment effects in other psychiatric disorders, such as MDD (Cash, Cocchi, Lv, Fitzgerald, & Zalesky, 2021; Cash et al., 2019; Fox, Buckner, White, Greicius, & Pascual-Leone, 2012a; Fox et al., 2013; Ning, Makris, Camprodon, & Rathi, 2019; Weigand et al., 2018); see online Supplementary Figure S8 for an illustration of the individual variability of the dlPFC voxel with peak connectivity to the anterior caudate among patients in the current sample. Individualized targets based on rsfMRI connectivity are still relatively novel and require further validation, but may provide a means through which to improve clinical outcomes among patients with AN.

Altered connection strength between the anterior caudate and dlPFC could stem from abnormal resting-state BOLD signal within the anterior caudate or could reflect broader, abnormal anterior caudate connectivity across multiple brain regions. To explore each of these possibilities, we examined group differences in amplitude of low-frequency fluctuations (ALFF), a proxy of BOLD signal intensity, and node centrality (using IC and GCOR) of the anterior caudate. We found no group differences in any of these measures, suggesting that on average, the anterior caudate's regional intensity and its connection strength to other areas of the brain is comparable between patients with AN and HC. These results point to some degree of specificity, such that abnormalities in anterior caudate connection strength may be specific to dorsal fronto-striatal circuitry. Alternatively, abnormalities in anterior caudate connection strength with brain regions other than the dlPFC may be obscured by heterogeneous connectivity such that some regions have increased connectivity while others are reduced, resulting in no group difference, on average.

Exploratory analyses of connectivity between the right anterior caudate and each of the regions with differential connection strength between AN and HC (right dlPFC, left SFG/MFG, right occipital cortex) found no significant correlations between connectivity and psychological features of AN after correcting for multiple comparisons. This may be due to our sample size: recent research suggests that sample sizes in the thousands may be necessary to reliably detect brain-behavior associations due to small effect sizes (Marek et al., 2022); therefore, even with the relatively large sample in this study, we may still be underpowered to detect stable brain-behavior associations (Grady, Rieck, Nichol, Rodrigue, & Kennedy, 2021; Yarkoni, 2009).

This study also found heightened connectivity between the right anterior caudate and right occipital cortex among patients with AN. Prior studies in rsfMRI have identified decreased connectivity within visual networks and between visual and sensorimotor networks among patients with AN (Amianto et al., 2013; Favaro et al., 2012; Phillipou et al., 2016), which the authors suggested could underlie body image disturbance. Anatomical and functional connections between the anterior caudate and occipital lobe, specifically, have been shown in healthy individuals and are termed the visual cortico-striatal loop (Nasr & Rosas, 2016; Seger, 2013), though the function of this loop is still largely unknown and warrants additional investigation, particularly among clinical populations such as patients with AN.

This study has strengths and certain limitations. The use of Combat to harmonize data is a particular strength of the study, as it allowed for examination of rsfMRI data across different scanners to maximize power. Further, our sample was almost completely unmedicated, enhancing confidence that findings are not confounded by the effects of psychotropic medications. A limitation of this study is that it examined brain differences in patients currently ill with AN; therefore, decreased coupling between the dlPFC and dorsal striatum at rest among patients with AN could represent a biological vulnerability to the development or maintenance of AN, or could function as a biomarker of illness secondary to patients' malnourished state. Additionally, this study included females only, which limits the generalizability of findings. Future studies may wish to explore whether connectivity within these circuits is also relevant to males with AN.

The reduction in dorsal fronto-striatal connection strength among patients with AN may reflect an important marker of the disease state or mechanism and could be considered a potential target for brain stimulation treatments. Continuing to amass larger samples by pooling data from smaller studies, as done here, is critical to reaching the goal of full-powered studies, particularly among uncommon and difficult-to-treat disorders, such as AN. Identification of promising targets for neuromodulation is a priority due to the challenges in treatment of AN. Future investigations of differences in dorsal fronto-striatal resting-state functional connectivity among a large sample of weight-restored patients with AN could further clarify the role of these circuits in illness and recovery.

## Supporting information

Muratore et al. supplementary material Muratore et al. supplementary material

## Supplementary material

The supplementary material for this article can be found at https://doi.org/10.1017/S003329172400031X.
