# Supplementary material for: Reduced dorsal fronto-striatal connectivity at rest in anorexia nervosa
Source: Psychol Med. 2024 Mar 18;54(9):2200–9. doi: 10.1017/S003329172400031X (PMC11413358; doi:10.1017/S003329172400031X)
Supplement: Muratore et al. supplementary material [file S003329172400031Xsup001.docx]

**Supplementary Materials**

Table of Contents

[MRI Scan Parameters 1](#_Toc150720060)

[Clinical Characteristics of Patient Sample 2](#_Toc150720061)

[Harmonized Data Analyses 3](#_Toc150720062)

[Unharmonized Data Analyses 6](#_Toc150720063)

[GLM with IQ as covariate, *n* = 169 6](#_Toc150720064)

[GLM without IQ as covariate, *n* = 181 10](#_Toc150720065)

[Clinical Correlates 14](#_Toc150720066)

[Individual rsfMRI Targets 15](#_Toc150720067)

# MRI Scan Parameters

Below is a summary of MRI parameters by site (Table S1) and motion parameters, including Framewise Displacement and Global Signal Change by group (Table S2). There were no group differences in motion during scans. Six participants (3 AN, 3 HC) had a run excluded from analysis due to >25% outlier volumes.

| **Table S1:** Study scanning parameters by site. | | | | |
| --- | --- | --- | --- | --- |
|  | **Site 1** | **Site 2** | **Site 3** | **Site 4** |
| **MRI scanner** | 3T GE Signa | 3T Philips | 3T GE Signa | 1.5T Philips |
| **No. channels per coil** | 32 | 8 | 32 | 8 |
| **Number of slices** | 34 | 36 | 11 | 33 |
| **Single vs. Multi-band** | Single | Single | Multi | Single |
| **Number of volumes** | 155 | 240 | 394 | 150 |
| **Slice thickness (mm)** | 3.5 | 3.0 | 2.0 | 3.0 |
| **TR (ms)** | 2,000 | 2,000 | 850 | 2,000 |
| **TE (ms)** | 30 | 19 | 25 | 40 |
| **Number runs** | 2 | 1 | 2 | 2 |
| **Time/run (min:s)** | 05:00 | 08:06 | 05:35 | 05:00 |
| **Eyes closed/open** | Closed | Open | Open | Open |

| **Table S2:** Mean Framewise Displacement (FD) and Global Signal Change (GSC) by group. Six runs (3 AN, 3 HC) with > 25% outlier volumes were excluded from analyses. | | | |
| --- | --- | --- | --- |
|  | **HC** | **AN** | **Group Difference** |
| **Framewise Displacement** | .10 (.04) | .10 (.04) | *T*(1,180) = .28, *p* = .78 |
| **Global Signal Change** | .78 (.04) | .78 (.03) | *T*(1, 180) = -0.04, *p* = .97 |

**Clinical Characteristics of Patient Sample**

**a**

#

**b**

**c**

***Figure S1****. Clinical characteristics of patients with AN.* **a)** Body Mass Index in kg/m^2^. **b)** Duration of illness in years. **c)** EDE-Q Global Scores.

# Harmonized Data Analyses

1. Seed-based functional connectivity
   1. *Small-volume correction analyses.* Summary of findings from small-volume corrected seed-based functional connectivity analyses using harmonized data are included below (Table S3).

| **Table S3:** Results of general linear models (GLM) comparing left and right anterior caudate-dlPFC connectivity between HC and individuals with AN using harmonized data, controlling for age and IQ. No group differences in left anterior caudate-dlPFC connectivity. Compared to HC, individuals with AN exhibit significantly reduced right anterior caudate-dlPFC connectivity. | | | | | | |
| --- | --- | --- | --- | --- | --- | --- |
|  | **Left anterior caudate** | | | **Right anterior caudate** | | |
|  | *F* | *P* | η^2^p | *F* | *p* | η^2^p |
| **Group** | 2.51 | .115 | .02 | 9.45 | .002 | .05 |
| **Age** | .17 | .684 | <.01 | .55 | .458 | <.01 |
| **IQ** | .05 | .832 | .00 | .30 | .587 | <.01 |

- 1. *Whole-brain analyses.* Summary of findings from whole-brain seed-based functional connectivity analyses using harmonized data are included below (Table S4). Image depicting these findings are included in the main article (Figure 2).

| **Table S4:**  Results of exploratory whole-brain seed-based functional connectivity analysis comparing right anterior caudate connectivity between HC and individuals with AN using harmonized data, controlling for age an IQ (voxel-wise threshold p-uncorrected <.001, cluster-size p-FDR-corrected <.05). Results indicated a significant effect of group for right anterior caudate connectivity with frontal and occipital regions. | | | | | | | | | |
| --- | --- | --- | --- | --- | --- | --- | --- | --- | --- |
| **Seed** | **Anatomical location** | | **MNI (xyz)** | | | **Cluster size** | **Group Difference** | **p-FWE** | **p-FDR** |
| **Right anterior caudate** | Frontal Lobe | L Superior Frontal Gyrus/Middle Frontal Gyrus | -20 | +24 | +38 | 158 | HC > AN | .028 | .032 |
|  | Occipital Lobe | Left Cuneal Cortex | -16 | -86 | +22 | 148 | AN > HC | .038 | .032 |

- 1. *Sensitivity Analyses: Subtype Differences*


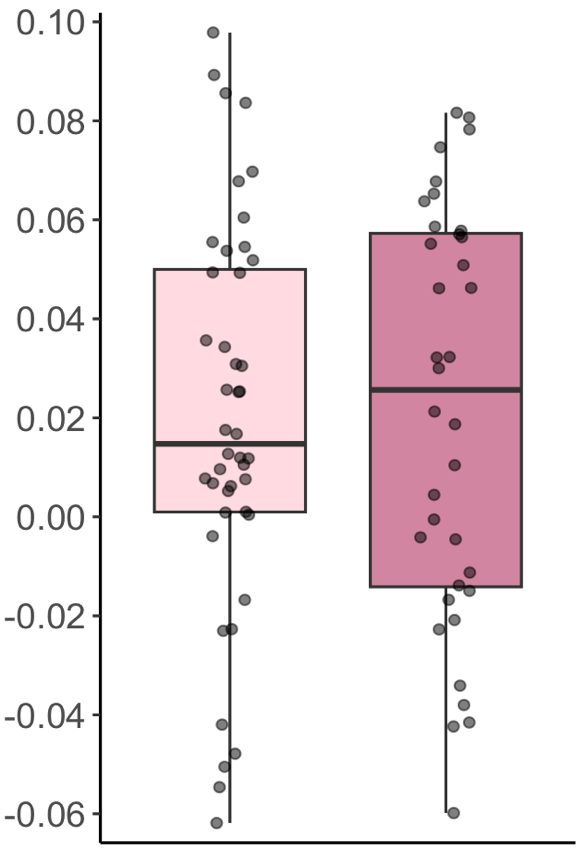


Right anterior caudate-dlPFC connectivity (z-score)

BP

R

*p* = .93

***Figure S2****. Results of sensitivity analysis comparing right anterior caudate-dlPFC connectivity between individuals with AN-BP (light pink) and AN-R (maroon). No significant group difference between subtypes, p = .93.*

- 1. *Sensitivity Analyses: Psychiatric Comorbidities:* The breakdown of current comorbidities in the sample was consistent with other samples of participants with AN. Thirty nine percent met criteria for a current anxiety disorder, 38% for a mood disorder, 8% for PTSD and 6% for OCD. Results of sensitivity analyses comparing HC and individuals with AN with no psychiatric comorbidities are included below.

Right anterior caudate-dlPFC connectivity (z-score)

HC

AN, no comorbidity

*****

*p = .002**


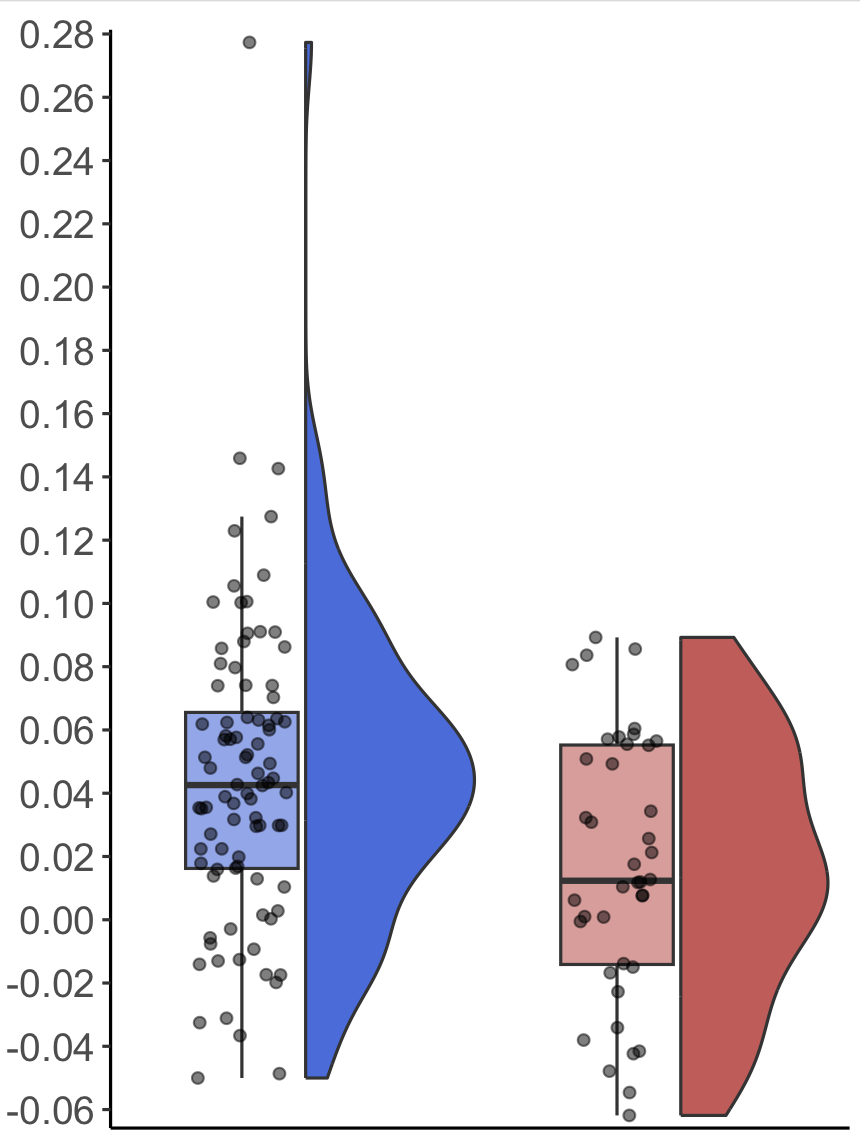


***Figure S3****. Results of sensitivity analysis comparing right anterior caudate-dlPFC connectivity between HC and individuals with AN with no psychiatric comorbidities (n = 45). Compared to HC, individuals with AN exhibit significantly reduced right anterior caudate-dLPFC connectivity.**Group difference remains significant when removing one outlier from HC group, *p* = .002

# Unharmonized Data Analyses

## **GLM with IQ as covariate, *n* = 169**

1. Seed-based functional connectivity
2. *Small-volume correction analyses.* Summary of findings from small-volume corrected seed-based functional connectivity analyses using unharmonized data are included below (See Figure S4, Table S5).

Left anterior caudate-dlPFC connectivity (z-score)

***Figure S4.*** *Top row:* *Left and right anterior caudate-dlPFC connectivity by group. . Group difference in right anterior caudate-dlPFC connectivity remains significant after removing outlier (n=1 HC), p = .002. Bottom row: Left and right anterior caudate-dlPFC connectivity by group and study.*

*****

*p = .002*

Right anterior caudate-dlPFC connectivity (z-score)

Left anterior caudate-dlPFC connectivity (z-score)

Right anterior caudate-dlPFC connectivity (z-score)

*
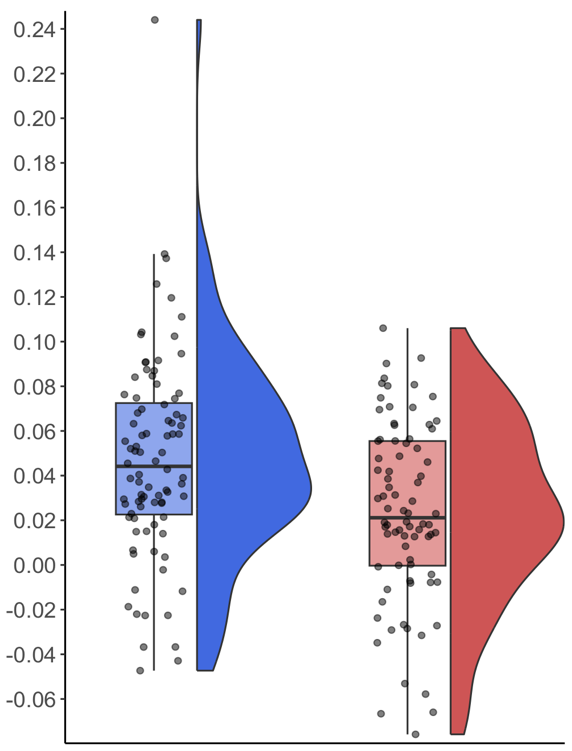

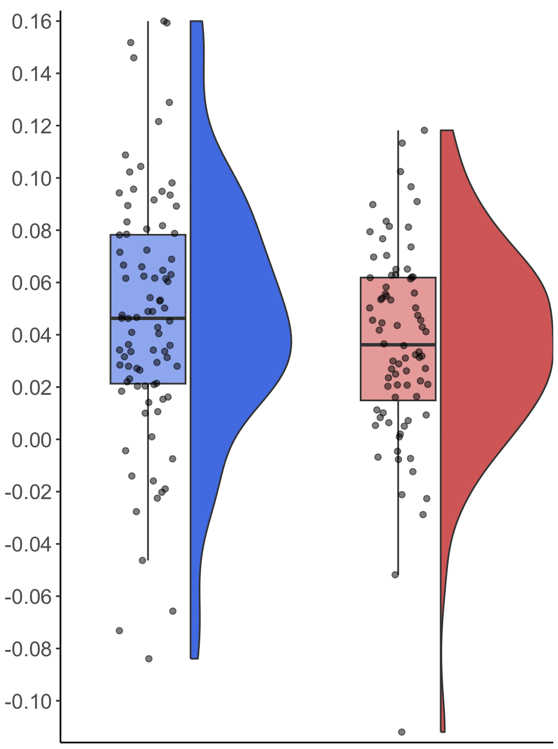
*


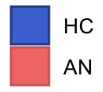


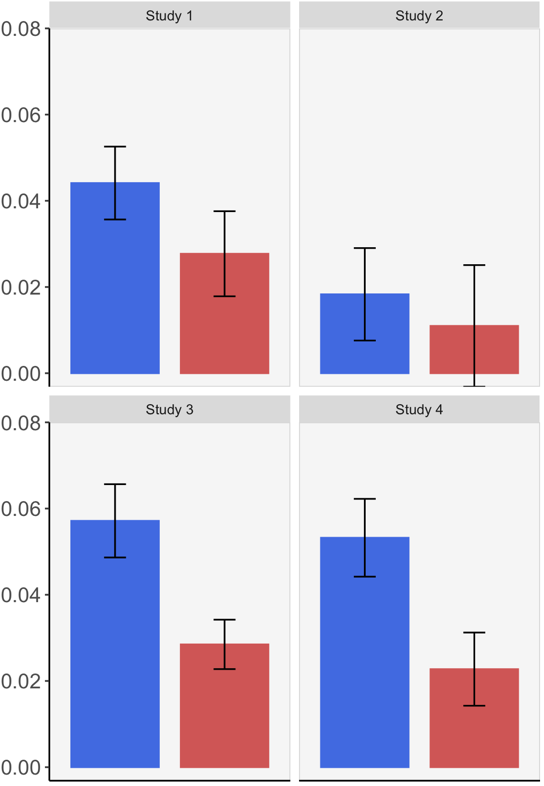

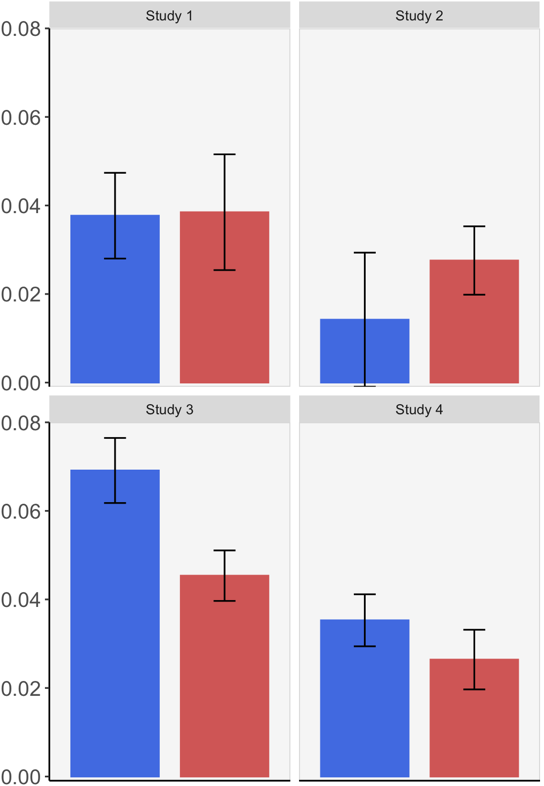


| **Table S5:** Results of GLM comparing left and right anterior caudate-dlPFC connectivity between HC and individuals with AN using unharmonized data. No group differences in left anterior caudate-dlPFC connectivity. Compared to HC, individuals with AN exhibit significantly reduced right anterior caudate-dlPFC connectivity, after controlling for age, site, IQ, and group by site interactions. | | | | | | |
| --- | --- | --- | --- | --- | --- | --- |
|  | **Left anterior caudate** | | | **Right anterior caudate** | | |
|  | *F* | *P* | η^2^p | *F* | *P* | η^2^p |
| **Group** | .28 | .601 | <.01 | 6.79 | .010 | .04 |
| **Site** | 5.09 | .002 | .09 | 2.97 | .034 | .05 |
| **Age** | .20 | .659 | <.01 | .30 | .588 | <.01 |
| **IQ** | .13 | .719 | <.01 | .24 | .624 | <.01 |
| **Group*Site** | 1.52 | .212 | .03 | .61 | .609 | .01 |

1. *Whole-brain analyses.* Summary of findings from whole-brain seed-based functional connectivity analyses using unharmonized data are included below (See Figure S2, Table S6).

***Figure S5.*** *Comparison of left and right anterior caudate whole-brain connectivity between HC and individuals with AN using unharmonized data, controlling for age, IQ, site, and including a group by site interaction term (voxel-wise height threshold p-uncorrected <.001, cluster-size p-FDR-corrected <.05, AN > HC = red; HC > AN = blue).* ***(a):*** *Results of seed-based functional connectivity using left anterior caudate as a seed.* ***(b)****; Results of seed-based functional connectivity using right anterior caudate as a seed.*

y = -9

x = -24

y = -31

z = 40

x = -22

z = 0

voxel-wise *p*_uncorr_ <.001, cluster-size *p*_FDR_< .05


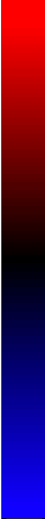


**HC > AN**

-5.00

5.00

*T*-value

1. **Left anterior caudate connectivity**
2. **Right anterior caudate connectivity**

**AN > HC**


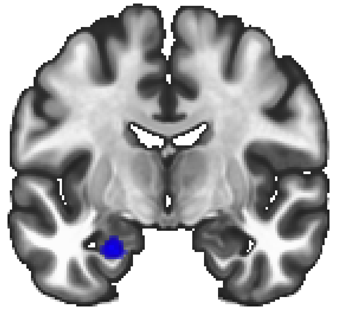

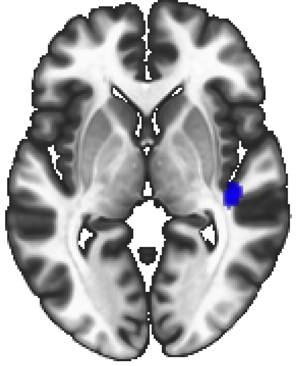

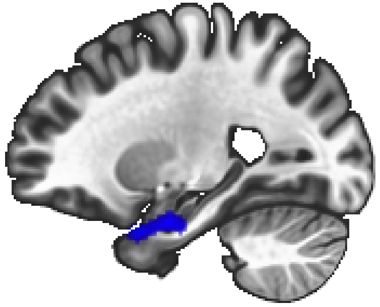

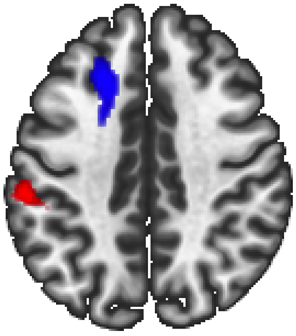

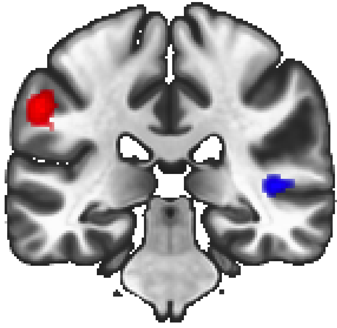

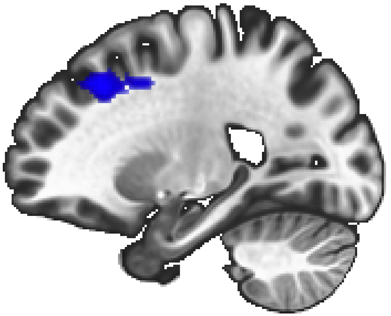


| **Table S6:**  Results of exploratory whole-brain seed-based functional connectivity analysis comparing anterior caudate connectivity between HC and individuals with AN using unharmonized data, controlling for age, IQ, site, and including a group by site interaction term (voxel-wise threshold p-uncorrected <.001, cluster-size p-FDR-corrected <.05). Results indicated a significant effect of group for left and right anterior caudate connectivity with frontal, temporal, and parietal regions. | | | | | | | | | | |
| --- | --- | --- | --- | --- | --- | --- | --- | --- | --- | --- |
| **Seed** | **Anatomical location** | | **MNI (xyz)** | | | **Cluster size** | **Group Difference** | **p-FWE** | | **p-FDR** |
| **Left anterior caudate** | Temporal Lobe | R Insula/Heschl’s Gyrus | +42 | -24 | +04 | 277 | HC > AN | .003 | .005 | |
|  |  | L Hippocampus/Amygdala | -24 | -06 | -24 | 226 | HC > AN | .009 | .008 | |
| **Right anterior caudate** | Frontal Lobe | L Superior Frontal Gyrus/Middle Frontal Gyrus | -20 | +24 | +40 | 407 | HC > AN | <.001 | .001 | |
|  | Temporal Lobe | R Insula/Heschl’s Gyrus | +44 | -30 | +04 | 191 | HC > AN | .021 | .019 | |
|  | Parietal Lobe | L Supramarginal Gyrus | -56 | -28 | +38 | 195 | AN > HC | .019 | .019 | |

1. Anterior caudate regional intensity

Summary of findings from anterior caudate regional intensity analyses using unharmonized data are included below (Table S7).

| **Table S7.** Results of general linear models (GLMs) examining effects of group (AN vs. HC) on amplitude of low-frequency fluctuation (ALFF) within the left and right anterior caudate, controlling for age, IQ, site, and group by site interactions. There were no group differences in either measure of left or right anterior caudate regional BOLD signal intensity, as measured by ALFF. | | | | | | | |
| --- | --- | --- | --- | --- | --- | --- | --- |
|  | **Left anterior caudate** | | | **Right anterior caudate** | | | |
| **Predictor** | *F* | *P* | η^2^p | *F* | *p* | η^2^p |  |
| **Group** | <.01 | .981 | .00 | .05 | .829 | .00 |  |
| **Site** | 247.14 | <.001 | .82 | 206.00 | <.001 | .80 |  |
| **Age** | 1.89 | .172 | .01 | 2.03 | .156 | .01 |  |
| **IQ** | .009 | .926 | .00 | .097 | .756 | <.01 |  |
| **Group*Site** | .39 | .764 | .01 | .39 | .762 | .01 |  |

1. Node centrality

Summary of findings from node centrality analyses using unharmonized data are included below (Table S8).

| **Table S8:** Results of GLM examining effects of group (AN vs. HC) on node centrality of left and right anterior caudate, controlling for age, IQ, site, and group by site interactions. There were no group differences in either measure of left or right anterior caudate node centrality. | | | | | | | |
| --- | --- | --- | --- | --- | --- | --- | --- |
|  |  | **Left anterior caudate** | | | **Right anterior caudate** | | |
| **Measure** | **Predictor** | *F* | *P* | η^2^p | *F* | *p* | η^2^p |
| **Intrinsic Connectivity (IC)** | Group | .04 | .846 | .00 | .18 | .674 | <.01 |
|  | Site | 186.93 | <.001 | .78 | 166.71 | <.001 | .76 |
|  | Age | .72 | .398 | <.01 | .29 | .594 | <.01 |
|  | IQ | .50 | .480 | <.01 | .44 | .508 | <.01 |
|  | Group*Site | 1.10 | .353 | .02 | .53 | .665 | .01 |
| **Global Correlation (GCOR)** | Group | .614 | .434 | <.01 | 1.20 | .275 | .01 |
|  | Site | 15.93 | <.001 | .23 | 13.23 | <.001 | .20 |
|  | Age | 2.26 | .134 | .01 | 2.43 | .121 | .02 |
|  | IQ | .33 | .565 | <.01 | .91 | .341 | .01 |
|  | Group*Site | .20 | .896 | <.01 | 1.44 | .233 | .03 |

## **GLM without IQ as covariate, *n* = 181**

1. Seed-based functional connectivity
2. *Small-volume correction analyses.* Summary of findings from small-volume corrected seed-based functional connectivity analyses using unharmonized data are included below (Figure S6, Table S9).

***Figure S6.*** *Top row: Left and right anterior caudate-dlPFC connectivity by group. Group difference in right anterior caudate-dlPFC connectivity remains significant after removing outlier (n=1 HC), p = .003. Bottom row: Left and right anterior caudate-dlPFC connectivity by group and study.*


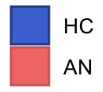


Left anterior caudate-dlPFC connectivity (z-score)

Left anterior caudate-dlPFC connectivity (z-score)

Right anterior caudate-dlPFC connectivity (z-score)

Right anterior caudate-dlPFC connectivity (z-score)

*****

*p = .001*


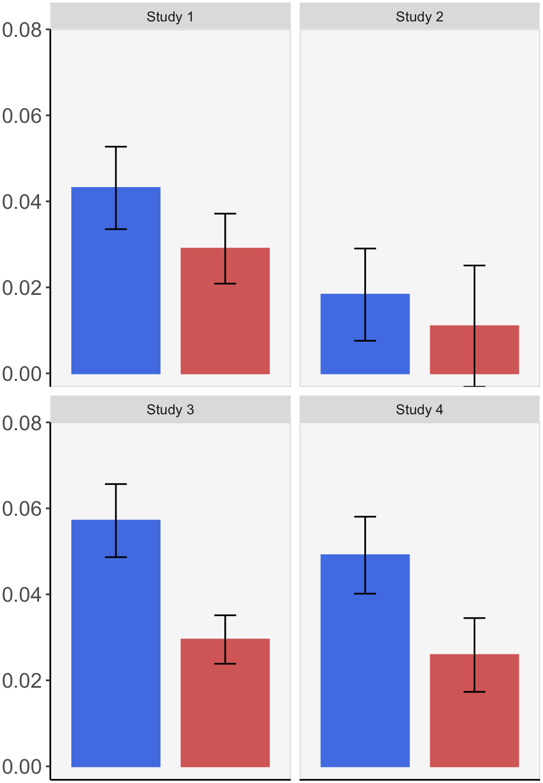

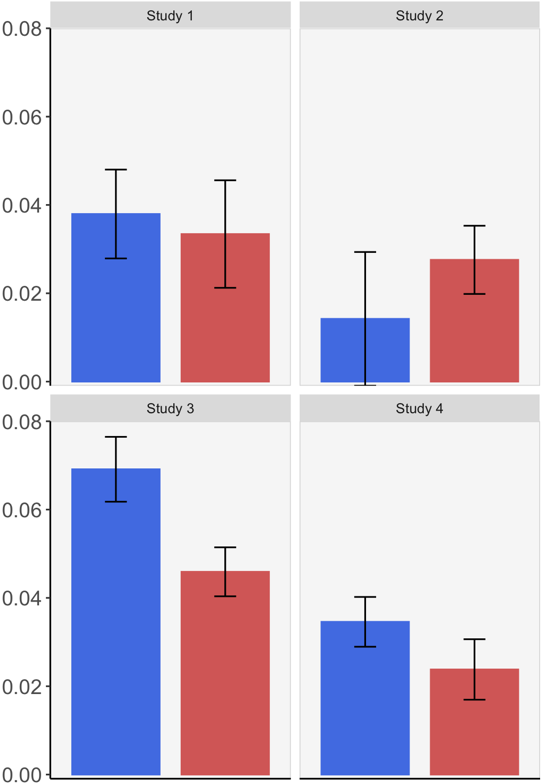


*
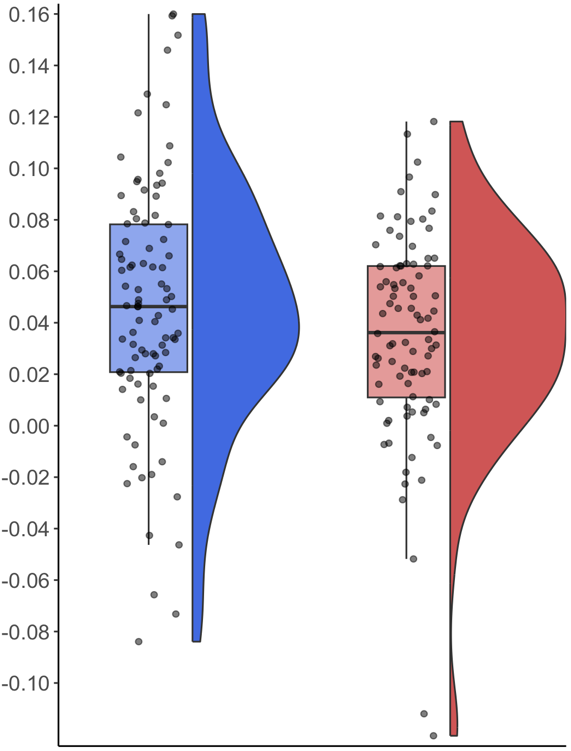

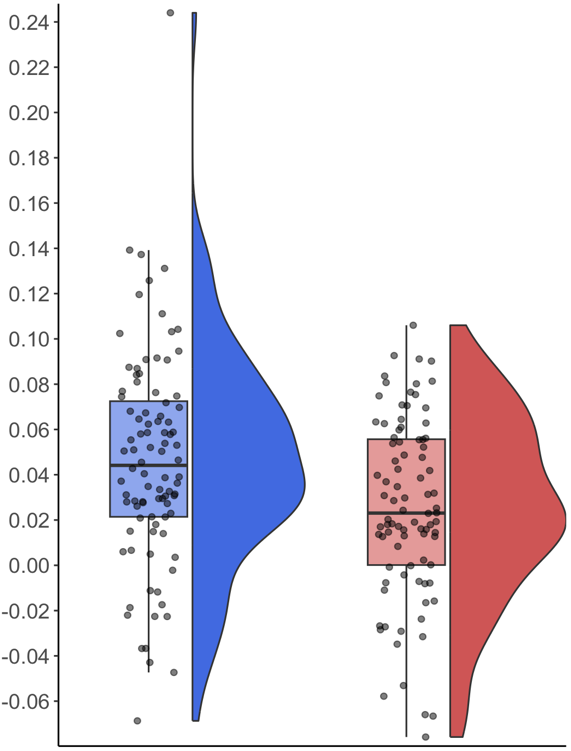
*

| **Table S9:** Results of GLM comparing left and right anterior caudate-dlPFC connectivity between HC and individuals with AN using unharmonized data. No group differences in left anterior caudate-dlPFC connectivity. Compared to HC, individuals with AN exhibit significantly reduced right anterior caudate-dlPFC connectivity, after controlling for age, site, and group by site interactions. | | | | | | |
| --- | --- | --- | --- | --- | --- | --- |
|  | **Left anterior caudate** | | | **Right anterior caudate** | | |
|  | *F* | *P* | η^2^p | *F* | *p* | η^2^p |
| **Group** | .84 | .360 | .01 | 6.36 | .013 | .04 |
| **Site** | 4.94 | .003 | .08 | 3.03 | .031 | .05 |
| **Age** | .74 | .390 | <.01 | .25 | .620 | <.01 |
| **Group*Site** | 1.17 | .322 | .02 | .50 | .685 | .01 |

1. *Whole-brain analyses.* Summary of findings from whole-brain seed-based functional connectivity analyses using unharmonized data are included below (Figure S4, Table S10).

***Figure S7.*** *Comparison of whole-brain connectivity to the left and right anterior caudate between HC and individuals with AN using unharmonized data, covarying for age, IQ, study, and including a group by study interaction term (voxel-wise height threshold p-uncorrected <.001, cluster-size p-FDR-corrected <.05, AN > HC = red; HC > AN = blue).* ***(a):*** *Results of seed-based functional connectivity using left anterior caudate as a seed.* ***(b)****; Results of seed-based functional connectivity using right anterior caudate as a seed.*

y = -5

x = -24

y = -30

z = 40

x = 45

z = -2

voxel-wise *p*_uncorr_ <.001, cluster-size *p*_FDR_< .05


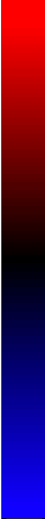


**HC > AN**

-5.00

5.00

*T*-value

1. **Left anterior caudate connectivity**
2. **Right anterior caudate connectivity**

**AN > HC**


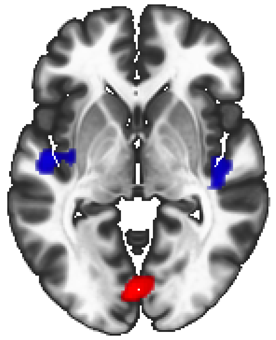

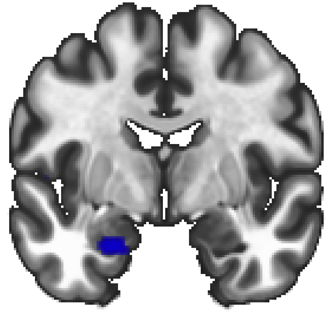

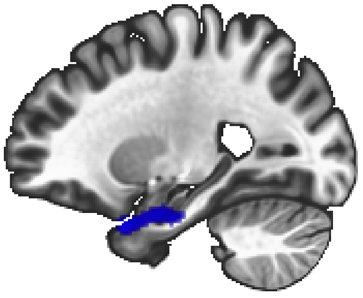

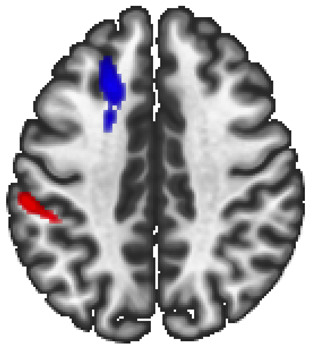

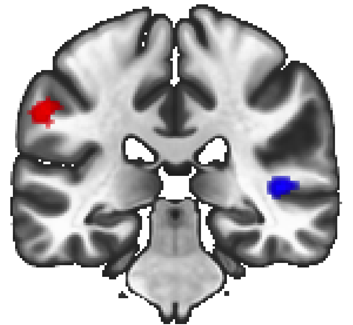

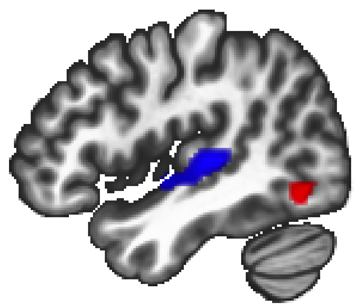


| **Table S10:**  Results of exploratory whole-brain seed-based functional connectivity analysis comparing anterior caudate connectivity between HC and individuals with AN using unharmonized data, controlling for age, site, and including a group by site interaction term (voxel-wise threshold p-uncorrected <.001, cluster-size p-FDR-corrected <.05). Results indicated a significant effect of group for left and right anterior caudate connectivity with frontal, temporal, parietal, and occipital regions. | | | | | | | | | | |
| --- | --- | --- | --- | --- | --- | --- | --- | --- | --- | --- |
| **Seed** | **Anatomical location** | | **MNI (xyz)** | | | **Cluster size** | **Group Difference** | **p-FWE** | | **p-FDR** |
| **Left anterior caudate** | Temporal Lobe | R Insula/Heschl’s Gyrus | +44 | -24 | +04 | 469 | HC > AN | <.001 | <.001 | |
|  |  | L Hippocampus/Amygdala | -26 | +06 | -30 | 286 | HC > AN | .004 | .002 | |
|  |  | L Insula/Heschl’s Gyrus | -48 | -12 | +00 | 284 | HC > AN | .004 | .002 | |
|  | Parietal Lobe | R Precuneus/Cingulate Gyrus | +10 | -46 | +06 | 191 | HC > AN | .028 | .012 | |
|  | Occipital Lobe | Bilateral Lingual Gyrus | -02 | -80 | -04 | 370 | AN > HC | <.001 | <.001 | |
| **Right anterior caudate** | Frontal Lobe | L Superior Frontal Gyrus/Middle Frontal Gyrus | -20 | +24 | +40 | 238 | HC > AN | .005 | .003 | |
|  | Temporal Lobe | R Insula/Heschl’s Gyrus | +44 | -26 | +04 | 351 | HC > AN | <.001 | .001 | |
|  | Parietal Lobe | L Supramarginal Gyrus | -42 | -40 | +30 | 256 | AN > HC | .003 | .003 | |
|  | Occipital Lobe | R Occipital Cortex | +48 | -62 | -14 | 148 | AN > HC | .046 | .021 | |

1. Anterior caudate regional intensity

Summary of findings from anterior caudate regional intensity analyses using unharmonized data are included below (Table S11).

| **Table S11:** Results of GLM examining effects of group (AN vs. HC) on amplitude of low-frequency fluctuation (ALFF) within the left and right anterior caudate, controlling for age, site, and group by site interactions. There were no group differences in either measure of left or right anterior caudate regional BOLD signal intensity, as measured by ALFF. | | | | | | | |
| --- | --- | --- | --- | --- | --- | --- | --- |
|  | **Left anterior caudate** | | | **Right anterior caudate** | | | |
| **Predictor** | *F* | *p* | η^2^p | *F* | *p* | η^2^p |  |
| **Group** | .01 | .909 | .00 | .13 | .720 | <.01 |  |
| **Site** | 288.82 | <.001 | .83 | 243.72 | <.001 | .81 |  |
| **Age** | 1.45 | .230 | .01 | 1.45 | .230 | .01 |  |
| **Group*Site** | .36 | .780 | .01 | .35 | .790 | .01 |  |

1. Node Centrality

Summary of findings from node centrality analyses using unharmonized data are included below (Table S12).

| **Table S12:**  Results of GLM examining effects of group (AN vs. HC) on node centrality of left and right anterior caudate, controlling for age, site, and group by site interactions. There were no group differences in either measure of left or right anterior caudate node centrality. | | | | | | | |
| --- | --- | --- | --- | --- | --- | --- | --- |
|  |  | **Left anterior caudate** | | | **Right anterior caudate** | | |
| **Measure** | **Predictor** | *F* | *p* | η^2^p | *F* | *p* | η^2^p |
| **Intrinsic Connectivity (IC)** | Group | .00 | .993 | .00 | .04 | .847 | .00 |
|  | Site | 221.90 | <.001 | .80 | 197.42 | <.001 | .78 |
|  | Age | 1.11 | .295 | .01 | .40 | .527 | <.01 |
|  | Group*Site | 1.44 | .234 | .02 | .57 | .635 | .01 |
| **Global Correlation (GCOR)** | Group | 1.92 | .167 | .01 | 2.48 | .117 | .01 |
|  | Site | 17.52 | <.001 | .23 | 14.76 | <.001 | .20 |
|  | Age | 1.82 | .179 | .01 | 2.03 | .156 | .01 |
|  | Group*Site | .05 | .984 | <.01 | .99 | .397 | .02 |

# Clinical Correlates

| **Table S13:**  Partial correlations between regions with group differences in connectivity and clinical variables among individuals with AN, controlling for age and IQ. No correlations survived Bonferroni correction. | | | | | |
| --- | --- | --- | --- | --- | --- |
|  |  | **EDE-Q Global Score** | **Duration of Illness** | **Buffet meal,**  **kcal consumed** | **Buffet meal,  %fat consumed** |
| **Right anterior caudate-Right dlPFC connectivity** | *r* | .26 | -.11 | -.12 | -.06 |
|  | *p*^uncorrected^  *p*^corrected^ | *.033*  *(.396)* | *.344*  *(1.00)* | *.464*  *(1.00)* | *.729*  *(1.00)* |
| **Right anterior caudate-Left SFG/MFG connectivity** | *r* | .15 | .04 | -.25 | -.30 |
|  | *p*^uncorrected^  *p*^corrected^ | *.225*  *(1.00)* | *.710*  *(1.00)* | *.116*  *(1.00)* | *.055*  *(.660)* |
| **Right anterior caudate-Right occipital cortex connectivity** | *r* | -.21 | -.09 | -.07 | .04 |
|  | *p*^uncorrected^  *p*^corrected^ | *.086*  *(1.00)* | *.412*  *(1.00)* | *.653*  *(1.00)* | *.813*  *(1.00)* |

# Individual rsfMRI Targets


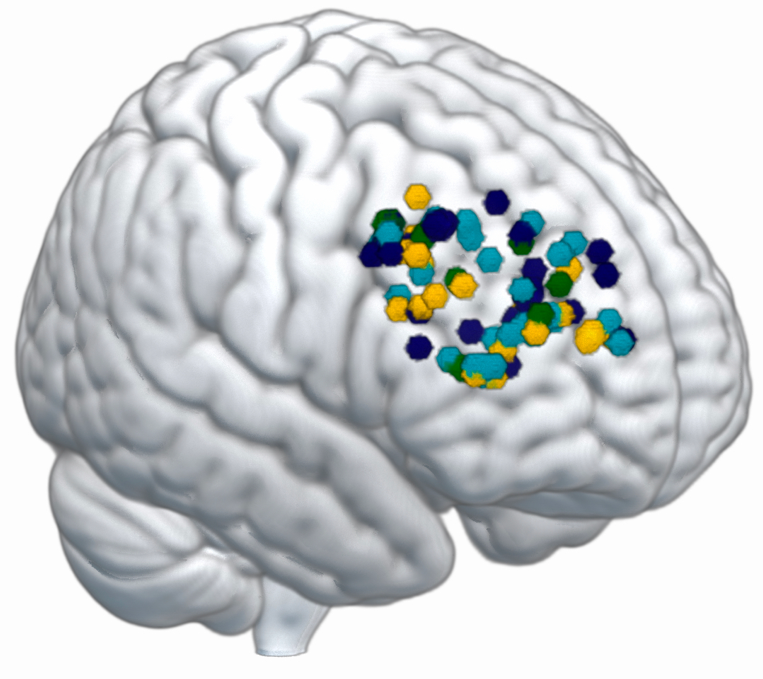


***Figure S8.*** *Illustration of potential individualized rsfMRI targets based on voxel of the dlPFC (with 3-mm sphere surrounding it) with strongest resting-state functional connectivity with the anterior caudate.*
